# A Comparative Electrochemical Behaviour Study and Analytical Detection of the p-Nitrophenol Using Silver Solid Amalgam, Mercury, and Silver Electrodes

**DOI:** 10.1155/2011/726462

**Published:** 2011-05-26

**Authors:** Djenaine De Souza, Lucia H. Mascaro, Orlando Fatibello-Filho

**Affiliations:** ^1^Campus de Patos de Minas, Instituto de Química, Universidade Federal de Uberlândia, Avenida Getúlio Vargas, 230 Centro, 38700-126 Patos de Minas, MG, Brazil; ^2^Departamento de Química, Universidade Federal de São Carlos, Rodovia Washington Luiz, Km 235, Caixa Postal 676, 13565-905 São Carlos, SP, Brazil

## Abstract

This work reports a comparative electrochemical behaviour study and p-nitrophenol analytical detection using silver solid amalgam, hanging dropping mercury, and silver electrodes. For this, square wave voltammetry was employed, where the analytical responses and the redox mechanisms could be compared for reduction processes of 4-nitrophenol by analysis of the voltammetric responses. The analytical performance of the electrode was evaluated and detection and quantification limits, recovery percentages, repeatability, and reproducibility for the silver solid amalgam and hanging dropping mercury electrodes presented similar values; the results presented for the silver electrode indicated worse analytical parameters than the other electrodes. The results indicate that the silver solid amalgam electrode can be considered a suitable tool and an interesting alternative for the analytical determination of 4-nitrophenol, as well as for the determination of other biological and environmentally interesting compounds that present analytical responses on mercury surfaces.

## 1. Introduction

The compound 4-nitrophenol (4-NP) is a nitro-aromatic that is frequently encountered as a product of the degradation or fabrication process of rubber chemicals, lumber preservatives, car exhausts, industrial wastes and also as the main degradation product of organophosphorous pesticides, such as fenitrothion and parathion. Due to the characteristics of its biorefractory compounds, it is considered a hazardous pollutant with toxic effects on human health and the environment [1].

As a result, in the last few years, numerous methodologies have been developed for the analytical determination of 4-NP in different samples. These procedures have involved the use of high-performance liquid chromatography [2], gas chromatography [3], capillary zone electrophoresis [4], and spectrophotometry with flow-injection analysis [5]. Meanwhile, various electroanalytical techniques also have been used with success as alternatives to chromatographic and spectroscopic techniques. 

For this, different solid surface electrodes, either bare or modified with different organic and inorganic compounds, have been designed to evaluate the analytical responses and to study the redox process mechanisms of the 4-NP [6–8]. Nevertheless, in this type of electrode, the renovation of the electrodic surfaces can be complicated due to the memory effects associated with the strong adsorption processes of organic compounds, which, in general, result in a decrease in the reproducibility of voltammetric responses.

Several works are based on processes occurring at Hg surfaces, such as in the hanging mercury drop electrode (HMDE). However, the highly toxic nature of mercury and other heavy metals makes the search for alternative electrode materials a necessity; for this, there is an increasing interest in the construction of solid electrodes using metallic amalgams, which are prepared by mixing a fine metal powder (Ag, Cu, Au, Bi, Sn, or Zn) with liquid mercury [9–11].

In general, solid amalgam electrodes are convenient electrodic surfaces because they are practically nontoxic, easily prepared, and mechanically stable. They also show long durability and allow for rapid surface pretreatment and simple electrochemical regeneration. The solid amalgam electrodes also present a solid and homogeneous surface, allowing easy renewal by mechanical polishing [12], and shows a high overpotential to the hydrogen evolution reaction [9–14]. Finally, as a major characteristic, these electrodes minimize any environmental contamination with mercury because the amount discarded in each analysis is minimal. 

Thus, the main goals of this work were to employ a silver solid amalgam electrode (AgSAE), to evaluate its efficiency in the study of electrochemical behaviour, and to establish an appropriate methodology for the analytical determination of 4-nitrophenol (4-NP). For this, the voltammetric responses obtained on the AgSAE were compared with similar results obtained using an HMDE and a silver electrode (AgE). 

With regard to the voltammetric technique, square wave voltammetry (SWV) was employed due to its extreme sensitivity in the detection of organic molecules. In addition, SWV allows valuable information concerning the electrochemical redox mechanisms to be obtained by the appropriate use of a well-developed theoretical model [15].

## 2. Experimental

### 2.1. Reagents and Equipment

All voltammetric measurements were carried out using an Autolab model PGSTAT 30 potentiostat from Metrohm-EcoChemie, which was controlled by a personal computer with GPES software (General Purpose Electrochemical System), version 4.9, from Metrohm-EcoChemie. 

A Denver Instrument Ultrabasic model pH-meter equipped with a 3.0 moL L^−1^ Ag/AgCl/KCl-glass combined electrode was used for adjusting pH values. Water of high purity (conductivity 1.0 *μ*S cm^−1^), purified by means of a Gehaka model OS20 LX Farma, was used to prepare all the solutions. 

A conventional cell with a three-electrode system was used in all experiments, which incorporated the Ag/AgCl/Cl^−^ 3.0 moL L^−1^ electrode as the reference electrode, a platinum wire as the auxiliary electrode, and a AgSAE, HMDE, or AgE as the working electrode.

The AgSAE was manufactured from amalgams produced according to procedure previously published [13]. A Metrohm model E410 hanging mercury drop electrode, with a drop area of 0.052 cm^2^, was used as one of the working electrodes. This electrode surface was renewed after each measurement was performed. A new drop was formed by dislodging the old one and extruding more triply-distilled mercury.

The AgE used was constructed from a 0.10 mm diameter silver wire (Goodfellow), where the wire was inserted into a piece of glass tube with approximately 0.30 mm internal diameter, which was later filled with Epoxy resin. After the Epoxy resin hardened, the AgE was polished using a mechanical polisher and glasspaper of different granulations. After this polishing procedure, the electrode was cleaned with water, and a smooth and homogeneous surface was observed.

All chemicals were of analytical-reagent grade. A 0.1 moL L^−1^ Britton-Robinson (BR) buffer was employed as the supporting electrolyte, and the pH was adjusted to the desired value by the addition of appropriate amounts of 1.0 moL L^−1^ NaOH stock solution.

A 1.0 × 10^−3^ moL L^−1^ stock solution of 4-NP from Merck (98% purity) was prepared daily by the dissolution of the appropriate quantity in ultra-pure water; the solution was stored in a dark flask and maintained in the refrigerator to avoid degradation.

### 2.2. Procedure for the Electrochemical Behaviour Study and Analytical Optimization

Before each day of experiments or after every delay longer than one hour, the AgSAE was activated according to the procedure previously described [9–13]. 

All measurements were carried out under ambient conditions. The appropriate solutions were transferred into the electrochemical cell. The optimization of the analytical procedure for SWV was then performed following a systematic study of the experimental parameters that affect the responses, such as the pH of the medium, the pulse potential frequency (*f*) related to the total pulse duration, the amplitude of the pulse (*a*), and the height of the potential step (Δ*E*_s_) or scan increment. 

The mentioned parameters were optimized in relation to the maximum value of peak current and the maximum selectivity (half-peak width). Before each experiment, the solutions were deaerated by bubbling nitrogen, and the electrochemical cell was kept under a nitrogen atmosphere throughout the experiments.

To accomplish the abovementioned, the working electrodes were placed in the measuring cell, which was filled with 10 mL of an electrolyte support solution containing a known concentration of 4-NP, and the experimental and voltammetric parameters were subsequently studied. In all experiments, the electrochemical cell was placed in a Faraday cage to minimize background noise. All parameters were optimized for the use of the AgSAE, HMDE, and AgE.

Analytical curves were obtained in pure electrolyte using the standard addition method. The standard deviation of the mean current (*S*_*b*_) measured at the reduction potentials of 4-NP for 10 voltammograms of the blank solution in pure electrolytes together with the slope of the straight line of the analytical curves (*s*) were used in the determination of the quantification and detection limits (QL and DL, resp.), according to guidelines recommended by IUPAC [16].

The recovery experiments were done in order to attest the methodology's efficiency. These experiments were carried out by adding a known amount of 4-NP to the supporting electrolytes followed by standard additions from the 4-NP, stock solutions and plotting the resulting analytical curves. All measurements were performed in triplicate. The recovery efficiencies (%*R*) were calculated considering the relation between the value of the concentration obtained by extrapolating the analytical curves of the corresponding spiked samples and the concentration previously added [17].

The precision and accuracy of methodology were tested with different standard solutions of 4-NP and the relative standard deviations (RSD) were calculated, considering the standard deviation of the mean current values obtained and the mean peak current values.

### 2.3. Application of Methodology

To attest the applicability of the proposed methodology, interfering effects were studied using water samples collected from two different points of the river Monjolinho, a local river in the city of São Carlos, Brazil. The sampling points, designated as sample 1 and sample 2, were known to show different characteristics with regard to pollution. Sample 1, located at the riverhead away from the city, is relatively free from urban or industrial pollution. Sample 2, a point halfway along the river length and situated in the city, has domestic and industrial sources of pollution. 

The samples were previously filtered in filter paper (12.5 cm *∅*, Vetec) and subsequently used to prepare the supporting electrolyte (BR buffer pH 7.0). Analytical curves were constructed similar to those for pure electrolyte. 

For the recovery curves, the samples were artificially spiked with 1.0 × 10^−6^ moL L^−1^ 4-NP solutions, and the recovery curves were constructed by the standard addition method, similar to the procedure used for the pure electrolyte. All experiments were performed in triplicate.

## 3. Results and Discussion

According to De Souza and coworkers [14], the AgSAE presents a globular structure with solid and compact surfaces, which enables the easy renewal of the electrode surface via mechanical polishing. Additionally, the presence of the prevailing form in the AgSAE (Ag_2_Hg_3_) with the nominal 30/70 (Ag/Hg : m/m) composition allows for the acquisition of good reproducibility in responses, even after mechanical polishing. For this, the analytical responses for 4-NP on the AgSAE were evaluated, and the results obtained were compared to similar results obtained using the HMDE and AgE. 

Several works have been previously published with reviews dealing the use of solid amalgam electrodes in the 4-NP determinations [18–21]. Despite that AgSAE has been intensively evaluated in amalgam surfaces, no works, to the best of our knowledge, reporting a comparative study in silver and traditional mercury electrodes and mainly the electrochemical behaviour study in silver and traditional mercury electrodes can be found in the available literature. For this, all voltammetric parameters were preliminary evaluated.

### 3.1. Electrochemical Behaviour Study and Voltammetric Optimization

**Figure 1:**
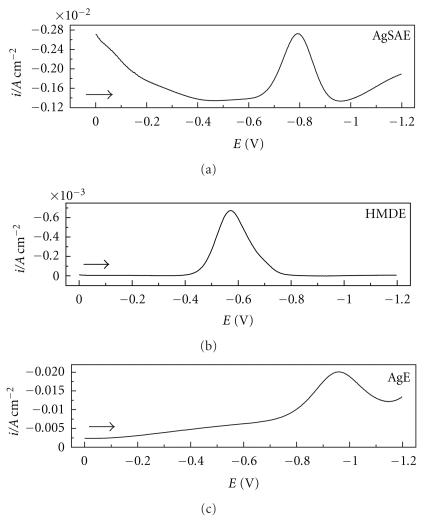
Square wave voltammograms for 1.05 × 10^−4^ moL L^−1^ 4-NP, using a 0.1 moL L^−1^ BR buffer solution at pH 7.0 and *f* = 100 s^−1^, *a* = 50 mV, and Δ*E*_s_ = 2 mV for the AgSAE, HMDE, and AgE. Currents are standardized by the electroactive area.

Preliminary experiments using SWV were realized for 4-NP using a sample of 0.1 moL L^−1^ BR buffer solution at pH 7.0 with 1.05 × 10^−4^ moL L^−1^ 4-NP solution, with parameters of *f* = 100 s^−1^, *a* = 50 mV, and Δ*E*_s_ = 2 mV. These experiments were used to determine the analytical responses to 4-NP of the AgSAE, HMDE, and AgE. The responses obtained are shown in Figure 1, where the presence of a single well-defined cathodic peak can be observed at around −0.56 V, −0.96 V, and −0.80 V for HMDE, AgE, and AgSAE, respectively. 

SWV experiments were also carried out to evaluate the type of redox process by analyses of the forward, reverse, and resultant components of current. The results demonstrated that the electrochemical behaviour of 4-NP presented characteristics of an irreversible electrode reaction controlled by the diffusion of the reagent, in close agreement with cyclic voltammetry results. Since the direct and reverse components of current are presented in the same direction, this indicates that the reactant and the product are both strongly adsorbed. 

**Figure 2:**
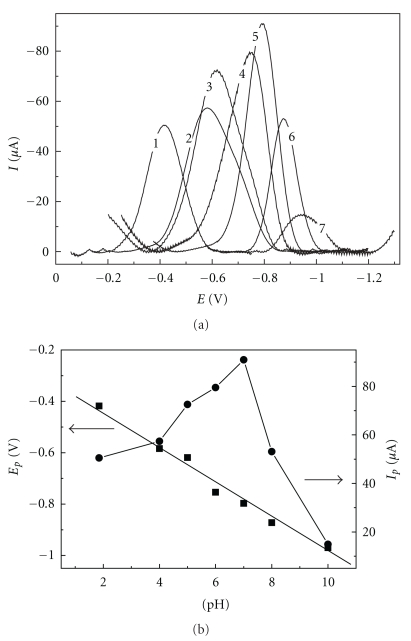
(a) Square wave voltammograms for 1.05 × 10^−4^ moL L^−1^ 4-NP, using a 0.1 moL L^−1^ BR buffer solution at different pH values and *f* = 100 s^−1^, *a* = 50 mV, and Δ*E*_s_ = 2 mV for the AgSAE, and (b) relationships between the pH values and the peak currents (right *y*-axis) and potentials (left *y*-axis).

A study about the effects of pH on the analytical responses for 4-NP on the AgSAE, AgE, and HMDE were also evaluated using the SWV technique. For this, a 0.1 moL L^−1^ BR buffer solution with varied pH between 2.0 and 10.0 was used with 1.05 × 10^−4^ moL L^−1^ 4-NP, *f* = 100 s^−1^, *a* = 50 mV and Δ*E*_s_ = 2 mV.  Figure 2 shows the responses obtained for 4-NP on the AgSAE at different pH values, where the insert indicates the responses for *I*_*p*_ and *E*_*p*_ as a function of the pH values used.

In all electrodes, it can be observed that the potential peaks were strongly pH-dependent, and the *E*_*p*_ shifts towards more negative values as the pH increases. This shift in potential with pH can be represented by the following equations (1), (2), and (3) for AgSAE, HMDE, and AgE, respectively,

(1) $$\begin{array}{c} E_{p}=-0.312-0.066\;\text{pH} \end{array},$$

(2) $$\begin{array}{c} E_{p}=-0.175-0.058\;\text{pH} \end{array},$$

(3) $$\begin{array}{c} E_{p}=-0.230-0.036\;\text{pH} \end{array},$$

where the potential values are given in volts. 

Equations (1) and (2) represent a straight line with a slope that is very close to the theoretical value predicted by the Nernst equation for an electrochemical reduction process involving the same number of protons and electrons (0.059 V/pH at 25°C). On the other hand, for use of the AgE, defined by (3), only one proton for two electrons can be involved in the electrode process. Considering the use of the AgSAE and HMDE, the results obtained are in accordance with previous work realized with other electrodic surfaces, such as boron doped diamond electrodes or modified electrodes [6–8].

Additionally, the relationships between the peak currents and pH values revealed that the peak currents present the maximum value at pH 4.00 for the HMDE and AgE, and for lower and higher pHs, the peak currents decreased sharply. For the AgSAE, the maximum current was observed at pH 7.0. A close analysis of this value shows that this pH is close to the pK_a_ value reported in the literature [7], confirming that the participation of protonation equilibrium precedes the electron transfer reaction. 

More detailed studies about the electrochemical behaviour of 4-NP for the AgSAE, HMDE, and AgE were realized using cyclic voltammetric experiments. For these experiments, the potential scan rates were varied from 10 to 200 mV s^−1^. For the responses obtained, it was observed that the *I*_*p*_ increases with the square root of the scan rates in all electrodes used; in addition, it was confirmed to be a diffusion-controlled process with the characteristics of an irreversible process, including the absence of anodic peaks in the reverse scans.

Additionally, the relationships between peak currents and the square root of the scan rates allowed the determination of the number of electrons related to the 4-NP reduction process for each electrode employed. This was determined using the Randles-Svecik equation for an irreversible process, according to the following equation [22]:

(4) $$I_{p}=\left(2.99\times 10^{5}\right)n{\left(\alpha n_{a}\right)}^{1/2}C_{r}^{*}D_{r}^{1/2}v^{1/2}.$$

The values used in this equation were 1.05 × 10^−6^ moL cm^3^ for concentration of 4-NP, a *D*_*r*_ of 9.19 × 10^−6^ cm^2^ s^−1^ [23], *α* of 0.5, and *αn*_*a*_ calculated by *αn*_*a*_ = 47.7/(*E*_*p*_ − *E*_*p*/2_), where *E*_*p*_ is the peak potential and *E*_*p*/2_ is the half-height potential. From this, it was possible to evaluate the number of electrons in the determinate step and the total electrons involved in redox process. As a result, the AgSAE, HMDE, and AgE implicated the transference of one electron in the determinate step, and the total number of electrons calculated was four. These values, for number of electrons, are in accordance with previous works employing other electrodic surfaces [7, 24]. 

The square wave voltammetry parameters were optimized for analytical and redox mechanism proposes for the use of all electrodes. As is well known, a variation in the frequency of application of pulse potential usually exerts a marked effect on the response of SWV. This effect thus provides a good criterion for the diagnosis and can be used to indicate any process of adsorption or reaction in solution or the reversibility or irreversibility of the electrochemical process [14]. 

For the AgSAE, HMDE, and AgE, it was observed that an increase in the *f* values was accompanied by an increase in *I*_*P*_. Additionally, a linear relationship was obtained between the *I*_*P*_ values and the square root of the *f* values, which, according to the theoretical model proposed by Lovric and coworkers [14] for SWV, may indicate diffusion-controlled electrode process. This was confirmed by the cyclic voltammetry experiments for all the electrodes, which also presented a linear dependence of the peak current on the square root of the scan rate and had a log *I*_*P*_ versus log *v* relationship with a slope very close to 0.5, a value expected for a diffusion controlled process [17]. Moreover, the inclination of the relationships between *I*_*P*_ and the square root of the *f* values supply a direct estimate for the values of the standard reaction rate constant (*k*) for a redox process, according to

(5) $$I_{P}=kf^{1/2}.$$

As a result, the values of *k* were 5.55 × 10^−6^  ± 4.40 × 10^−7^, 4.10 × 10^−6^  ± 1.57 × 10^−7^, and 2.63 × 10^−6^  ± 7.90 × 10^−8^ s^−1/2^ for the AgSAE, HMDE, and AgE, respectively. According to these values, it is possible to assume that the transfer kinetic of the process is faster for the AgSAE than for the HMDE or AgE. 

The influence of *f* on the *E*_*p*_ values was also evaluated, and the results showed a shift towards more positive values, varying linearly with the logarithmic value of *f* according to (13), which was developed for an irreversible redox process with adsorption complications:

(6) $$\frac{\Delta E_{\text{p}}}{\Delta \log\;f}=\frac{-2.3RT}{\alpha nF},$$

where *R* is the gas constant, *T* is the temperature, *α* is the electron transfer coefficient, *n* is the number of electrons only in the determining step of the process, and *F* is the Faraday constant. 

The slope of each curve was experimentally determined to be 29.47, 107.64, and 111.21 mV per decade for the AgSAE, HMDE, and AgE, respectively. Considering *α* = 0.5, using *T* as the room temperature, and substituting the known values of *R*, *F*, and the slope into the right-hand side of (5), *n* was calculated to be equal to four for the AgSAE, and one for the HMDE and AgE. 

Based on the previously observed results with the SWV parameters, in conjunction with the previous experiments of cyclic voltammetry (CV) and the literature reports [7, 24, 25], considering experiments with exhaustive electrolyses and determinations of products of reduction processes by spectroscopy techniques, it can be presumed that the 4-NP is irreversibly reduced in a 4-electron step. This process is probably due to a four-electron reduction of the nitro-group (R-NO_2_) to a hydroxylamine-group (R-NHOH), which is typical of the nitro-substituted compounds:

(7) $$\text{R-}{\text{NO}}_{2}+\;4{\text{e}}^{-}+\;4{\text{H}}^{+}\to \;\text{R-}\;\text{NHOH}+{\text{H}}_{2}\text{O}.$$

Considering the results from the HMDE and AgE for the determining steps, evaluated by CV and SWV experiments at pH 4.0, the transference of one electron is related to the slow step of the reaction, and three electrons are related to a fast step, defined by

(8) $$\text{R-}{\text{NO}}_{2}+{\text{e}}^{-}\to \text{R-}{\text{N}{\text{O}}_{2}}^{-}\;\left(\text{slow}\right),$$

(9) $$\text{R-}{\text{N}{\text{O}}_{2}}^{-}+3{\text{e}}^{-}+4{\text{H}}^{+}\to \text{R-}\;\text{NHOH}+{\text{H}}_{2}\text{O}\;\left(\text{fast}\right).$$

Upon studying the influence of the variation in amplitude (*a*), it was experimentally observed that *I*_*p*_ varied proportionally to *a*, up to 50 mV, without any observed shift in *E*_*p*_ or in the half-peak width. After 50 mV, the half-peak width showed an increase, suggesting a loss of sensibility of the method, considering the use of three electrodes. Following this observation, it was concluded that the best value of *a* for the detection of 4-NP was 50 mV.

Furthermore, a linear relationship can be observed between *I*_*p*_ and the *a* values, which can be employed to determine the concentration of the adsorbed species on the electrode surface, according to

(10) $$I_{p}=\left(5\pm 1\right)10^{2}A\alpha n^{2}Ff\Delta E_{s}\Gamma a,$$

where Γ is the adsorbed species concentration, and all terms represent parameters previously presented in this paper. From this, the Γ values calculated were 3.02 × 10^−7^, 6.29 × 10^−7^, and 1.62 × 10^−6^ moL/cm^2^ for the AgSAE, HMDE, and AgE, respectively. These results are indicative that 4-NP is less adsorptive for the AgSAE and that the greatest adsorptive process occurs for the AgE. This assumption is confirmed by fact that for the use of AgE, a step to clean the electrodic surfaces is necessary between each experiment, which increases the difficulty for analytical reproducibility.

According to the theoretical aspects of the SWV developed by Lovric and coworkers [15], for a totally irreversible redox process, the variation in half-peak width (Δ*E*_*p*/2_) of the SWV responses is dependent on the *αn* products, for *a* > 20 mV, according to

(11) $$\Delta E_{p/2}=\frac{\left(63.5\pm 0.5\right)}{\alpha n}.$$

Considering that *α* = 0.5, the calculated values for Δ*E*_*p*/2_ are given by 127/*n*. For 4-NP on the AgSAE, HMDE, and AgE, the Δ*E*_*p*/2_ values observed were 133, 117, and 121 mV, respectively. The difference between the observed and calculated values occurs because the system redox is influenced by the adsorption process and the *α* value is not exactly 0.5. 

For the scan increment, the increase in this value will also increase the signal and the sensitivity of the technique. However, for large values of Δ*E*_*s*_, a widening of the peaks may occur, thus diminishing the resolution of the analysis. For this reason, Δ*E*_*s*_ was evaluated for the reduction of 4-NP for the AgSAE, HMDE, and AgE. The results obtained show that the increase in Δ*E*_*s*_ does not have any influence on the peak potentials. In this case, an increase in Δ*E*_*s*_ results in an increase in *I*_*p*_, but no linear relationship was observed. As a result, in subsequent experiments, a value of Δ*E*_*s*_ = 2 mV was adopted.

### 3.2. Comparison between Analytical Sensitivities

Using the above optimized parameters, linear calibration curves were obtained for 4-NP on the three electrodes. For this, aliquots from the stock 4-NP solution were consecutively added to the electrochemical cell containing 10 mL of the electrolyte. The SWV responses were recorded for a concentration range between 5.51 × 10^−7^ and 4.86 × 10^−6^ moL L^−1^ for the AgSAE and HMDE and between 1.62 × 10^−5^ and 1.61 × 10^−4^ moL L^−1^ for the AgE. 

**Figure 3:**
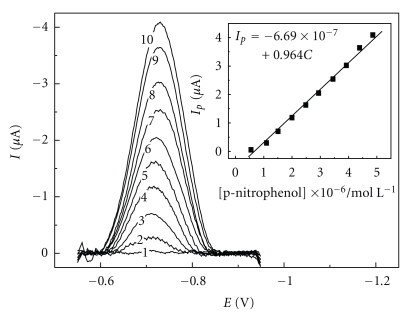
Square wave voltammograms obtained for the reduction of 4-NP for the AgSAE in 10 mL of BR buffer (pH 7.0), *f* = 100 s^−1^, *a* = 50 mV, and Δ*E*_*s*_ = 2 mV, with additions of 0.55 (1); 1.10 (2); 1.51 (3); 2.00 (4); 2.49 (5); 2.94 (6); 3.45 (7); 3.92 (8); 4.39 (9); 4.86 (10) ×10^−6^ moL L^−1^ 4-NP solutions. The insert shows the analytical curve obtained.

For the AgSAE, HMDE, and AgE, the square wave voltammograms presented linear relationships between the peak currents and the concentrations added.  Figure 3 shows the square wave voltammograms obtained with the AgSAE, and the insert presents the analytical curve obtained. 

The linearity between the densities of the peak currents and the added concentrations of 4-NP for the AgSAE, HMDE, and AgE are described by equations (12), (13) and (14), respectively,

(12) $$\begin{array}{c} i\;\left(\mu A/\text{c}{\text{m}}^{2}\right)=\;-1.99+1.674\times 10^{7}\;C\;\left(\text{moL}/\text{L}\right), \end{array}$$

(13) $$\begin{array}{c} i\;\left(\mu A/\text{c}{\text{m}}^{2}\right)=\;-0.79+1.801\times 10^{7}\;C\;\left(\text{moL}/\text{L}\right) \end{array},$$

(14) $$\begin{array}{c} i\;\left(\mu A/\text{c}{\text{m}}^{2}\right)=\;-1.72+1.908\times 10^{6}\;C\;\left(\text{moL}/\text{L}\right) \end{array}.$$

As these curve exhibited a negative interception, the presence of random errors was evaluated with the statistic test, *t*-test [26], and the calculated *t* values indicated that the negative interception was free from random errors. Besides, the analytical *sensitivities*, defined by the inclination of the analytical curves, for the AgSAE and HMDE presented similar values, indicating the similar *sensitivities* for both electrodes; meanwhile, for the AgE, the analytical curve inclination presented lower values, indicating that the AgE promotes responses with lower sensibilities than those obtained with the AgSAE and HMDE.

The detection (DL) and quantification limits (QL) were obtained for the experimental conditions and criteria presented in the *Experimental Section* for all electrodes employed, and Table 1 shows the experimental results obtained. This table shows the correlation coefficients (*r*), which determine the degree of linearity of the relationship between the concentration of 4-NP and the peak current (without considering the electroactive area), the standard deviations of the arithmetic mean of ten blank solutions (*S*_*b*_), the slope of the working curves (*s*), the detection limits (DL), and the quantification limits (QL). 

The values for the DL and QL obtained using the AgSAE and HMDE for the 4-NP reduction process presented very similar values, and these values were very close to those obtained previously for the HMDE or other electrodic surfaces [6, 7, 19]. For the AgE, the values for the DL and QL indicated that its surface is very inconvenient for the determination of 4-NP, due to the low sensitivity and, principally, to the difficulties related to the reproducibility of the analytical responses, which was caused by the strong adsorptive process of 4-NP. 

Recovery experiments were also performed using the AgSAE and HMDE in order to evaluate and compare the efficiency of each electrode. For this, pure electrolyte was composed of 0.10 moL L^−1^ BR buffer solutions, with the pH adjusted to 7.0 for the AgSAE and 4.0 for the HMDE; the pure electrolyte was then spiked with 1.05 × 10^−6^ moL L^−1^ 4-NP, followed by the addition of the standard solutions. The recovery percentages were used to evaluate and quantify the 4-NP that was added. In this way, the efficiency of each electrode could be determined. 

The recovery percentages obtained were around 94.95 ± 0.95% and 97.88 ± 0.89% for the AgSAE and HMDE, respectively, indicating that this methodology can be successfully applied for the analytical determination of 4-NP. 

The precision and accuracy of the analytical responses obtained for all proposed electrodes were evaluated by the experiments for reproducibility and repeatability. The reproducibility was evaluated on different days, using five different solutions containing 1.02 × 10^−6^ moL L^−1^ 4-NP for the AgSAE and HMDE and 1.06 × 10^−5^ moL L^−1^ 4-NP for the AgE. The RSD value obtained for *n* = 5 was lower than 2.00% for the AgSAE and HMDE (1.95% and 1.49%, resp.) but larger than 5.00% for the AgE.

The repeatability was evaluated ten times in the same solution containing 1.02 × 10^−6^ moL L^−1^ 4-NP for the AgSAE and HMDE and 1.06 × 10^−5^ moL L^−1^ 4-NP for the AgE. The RSD value obtained for *n* = 10 also presented values lower than 2.00% for the AgSAE and HMDE (1.60% and 1.25%, resp.).

### 3.3. Application to Natural Samples Water

To test the applicability of the AgSAE in complex samples, the methodology was employed for the construction of analytical curves and evaluation of recovery efficiencies for experiments using two natural water samples. These two natural water samples contained different level of contamination, which were determined by measurements of the biochemical oxygen demand (BOD) and chemical oxygen demand (COD). 

The water samples were used as collected as the solvent in preparing the BR buffer at pH 7.0. This procedure was aimed at identifying any influence of the matrix components on the analyses and detection of 4-NP for the AgSAE. It is important to highlight that prior to the introduction of the first aliquot of 4-NP, the electrolyte was free from any signs of 4-NP; consequently, the level of any possible contamination by nitro-compounds was below the detection limit of the proposed methodology.

**Figure 4:**
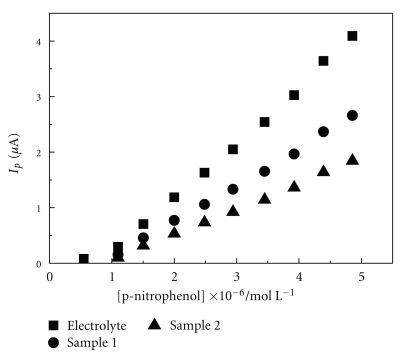
Analytical curves for the determination of 4-NP for the AgSAE obtained for (■) pure electrolyte, (●) natural water sample 1, and (▲) natural water sample 2 (*f* = 100 s^−1^, *a* = 50 mV, and Δ*E*_*s*_ = 2 mV).

The analytical curves for 4-NP on the AgSAE were evaluated using the natural samples water and were compared to the calibration curves obtained using pure water electrolyte. From these, it was possible to observe a reduction in the value of the slopes, indicating a loss of sensitivity in the analytical methodology, as shown in Figure 4. This loss of sensitivity must be related to the amount of organic matter present in the water samples, as indicated by the estimates of BOD and COD. 

The BOD and COD parameters are related to the relative oxygen requirements in the biochemical and chemical degradation, respectively, of organic materials and inorganic materials present in the samples, which are susceptible to oxidation. In practice, the COD values are slightly higher than the BOD values; thus, it can be inferred that the higher BOD and COD values obtained indicate a greater amount of organic matter present in the sample [27].

Recovery experiments were also performed to evaluate the interference of organic and inorganic components of the natural water samples on the reduction of 4-NP for the AgSAE. Recovery curves for the samples spiked with 1.0 × 10^−6^ moL L^−1^ 4-NP were obtained by the standard addition method. The results of the present study, shown in Table 2, depict the inverse dependence of the peak current on the amount of organic matter present in different samples. These results are shown to be in a suitable range for analytical applications where the acceptable values are in the interval between 70% and 130% [28].

## 4. Conclusions

The utilization of AgSAE minimized any environmental contamination with mercury because the amount discarded in each analysis is practically insignificant. The use of SWV was faster and more sensitive than other conventional techniques and to make possible to evaluate the electrochemical redox process that occurred for 4-NP on AgSAE.

The memory effects associated with the strong adsorption processes of 4-NP were unravelled by surface conditioning potential, which was sufficient to promote a total renewal thus yielding a new and reproducible surface. This extra advantage lowers the time used in the analysis, hence resulting in improvement in analytical sensibility and in the reproducibility of the responses. 

So, the results presented in this work proved that the AgSAE can be considered a suitable tool in the analytical determination and in the electrochemical behaviour study of the 4-NP, as well as other biological and environmentally interesting compounds that present analytical responses on mercury surfaces.

## Figures and Tables

**Table 1:** Analytical parameters for the determination of 4-NP on AgSAE, HMDE, and AgE.

Parameters	AgSAE	HMDE	AgE
*r*	0.9974	0.9970	0.9635
*S*_*b*_ (A)	1.724 × 10^−8^	2.186 × 10^−8^	5.978 × 10^−8^
*s* (A/moL L^−1^)	0.997	1.018	0.075
DL (moL L^−1^)	5.190 × 10^−8^ (7.22 *μ*g L^−1^)	6.442 × 10^−8^ (8.96 *μ*g L^−1^)	2.391 × 10^−6^ (332.64 *μ*g L^−1^)
QL (*μ*g L^−1^)	1.730 × 10^−8^ (24.66 *μ*g L^−1^)	2.147 × 10^−7^ (29.87 *μ*g L^−1^)	7.971 × 10^−6^ (1108.80 *μ*g L^−1^)
Rec. (%)	94.95 ± 0.95	97.88 ± 0.89	—

**Table 2:** Parameters from the analytical curves for 4-NP for the AgSAE in pure electrolyte and different natural water samples.

Parameter	Electrolyte	Sample 1	Sample 2
*r*	0.9974	0.9979	0.9955
*s* (A/moL L^−1^)	0.997 ± 0.026	0.657 ± 0.010	0.456 ± 0.074
Recovery (%)	94.95 ± 0.95	85.96 ± 1.79	72.28 ± 2.65
BOD (mg L^−1^)	—	6.0	12.0
COD (mg L^−1^)	—	19.0	33.0
